# Evaluation of Mechanical Anisotropy of New Polycarbonate Through Process Parameter Optimization in Material Extrusion

**DOI:** 10.3390/ma18245511

**Published:** 2025-12-08

**Authors:** Jaemin Han, Seongjun Kim, Ji Eun Lee, Yong Son, Seong Je Park

**Affiliations:** 1 School of Mechanical Engineering, Gyeongsang National University, 501 Jinju-daero, Jinju-si 52828, Gyeongsangnam-do, Republic of Korea; 2 Korea Additive Manufacturing Innovation Center (KAMIC), Korea Institute of Industrial Technology (KITECH), 113-58, Seohaean-ro, Siheung-si 15014, Gyeonggi-do, Republic of Korea

**Keywords:** additive manufacturing, material extrusion, tensile strength anisotropy

## Abstract

The parts fabricated using the material extrusion (MEX) process exhibit anisotropic mechanical properties depending on the build orientation, primarily due to incomplete interlayer bonding. In this study, a new polycarbonate (PC) is adopted, and the anisotropic behavior of parts produced from this material is quantitatively investigated in MEX. First, we examine the deformation behavior of the material extruded (MEXed) parts according to chamber temperature. Additionally, we evaluate the surface roughness of MEXed parts as a function of the nozzle temperature. Finally, to identify the tensile strength anisotropy, tensile tests are conducted on MEXed specimens in two deposition directions using three nozzle temperatures that produced superior surface roughness. As a result, a PC adopted in this study exhibits relatively low tensile strength anisotropy, indicating that it is well suited for the MEX. Overall, this study not only provides a systematic procedure for optimizing the process parameters when adapting a new polymer to MEX, but also offers practical guidance for evaluating tensile strength anisotropy to determine whether the material is suitable for MEX.

## 1. Introduction

Recently, additive manufacturing (AM) technology has emerged as an innovative manufacturing process capable of producing complex and precise three-dimensional components more efficiently and flexibly than traditional subtractive machining or injection molding [1,2]. AM provides designers with high geometric freedom, reduces material waste, and enables customized production. For these reasons, AM applications are expanding across various industries, including aerospace, medical devices, automotive, molds, and electronics [3,4].

Among the AM techniques, material extrusion (MEX) is characterized by relatively simple equipment, low-cost raw materials, and compatibility with a wide range of polymer-based materials and composites [5]. Due to these advantages, MEX has been widely adopted in both research and industrial applications [6]. However, samples and components fabricated via MEX often exhibit anisotropic mechanical properties, depending on the build or print orientation, which presents a critical challenge [7]. This anisotropy arises because the interlayer bonding strength varies with the layer orientation, ultimately hindering structural reliability and uniform material properties [8]. Therefore, studies aimed at minimizing mechanical anisotropy in MEX processes are essential.

Previous research has analyzed the anisotropic behavior of material extruded (MEXed) from various perspectives. Studies on nylon and short-fiber-reinforced composites demonstrated that tensile strength could increase up to twofold depending on the alignment between the print path and the loading direction [9]. Other research has shown that adjusting the nozzle temperature to enhance interlayer bonding reduces the strength difference between the longitudinal and transverse directions [10]. The application of carbon fiber-reinforced thermoplastic composites allowed the mitigation of directional differences in mechanical properties through fiber orientation control [11]. Additionally, optimizing infill patterns and build orientation was reported to homogenize internal stress distribution and reduce anisotropic behavior [12]. Furthermore, the comprehensive process conditions, such as nozzle temperature, layer height, raster angle, infill rate, and post-processing, were compared in terms of the mechanical properties of neat polymers [13]. Regarding polycarbonate (PC), previous studies have mainly focused on specimen types, build orientations, and MEX processing time in terms of neat PC [14].

However, quantitative studies focusing on interlayer adhesion and directional dependence from a material development perspective are still limited. To address this, the present study adopts a new PC suitable for MEX with superior interlayer bonding characteristics. To establish optimal MEX conditions for PC, this study first investigates the effects of chamber and nozzle temperatures on deformation and surface quality, respectively. Based on the nozzle temperatures that yielded superior surface roughness, tensile specimens were then MEXed in two deposition directions to assess the resulting tensile strength anisotropy. The overall experiments in this study suggest that the adopted PC exhibits low tensile strength anisotropy, indicating its suitability for MEX.

## 2. Methodology

### 2.1. Material

The PC (3DP-3A27R20, Samyang, Daejeon-si, Republic of Korea) used in this study was formulated by blending 60% fossil PC (Trirex 3027 IR, Samyang, Republic of Korea) with an EAA-type (ethylene-acrylic acid) compatibilizer incorporated at 10–15 wt% of the total blend, 20% bio PC (Durabio D7340 IR, Mitsubishi Chemical, Tokyo, Japan), and 20% recycled PC (PC LPC103 B07, Luckyenpla, Chilgok-gun, Republic of Korea). For the comparison of surface roughness and tensile properties, PC (PC10, Stratasys, Eden Prairie, MN, USA) and ABS (ABS Plus, Stratasys, Eden Prairie, MN, USA) were used.

### 2.2. MEX Process

For the MEX of 3DP-3A27R20, an optimized MEX machine was used [15]. The chamber temperature and nozzle temperature were varied from 40 °C to 150 °C and from 230 °C to 300 °C, respectively. The other process parameters of the layer thickness, nozzle diameter, and nozzle speed were set to values of 0.2 mm, 0.4 mm, and 40 mm/s, respectively. The Fortus 450 mc (Stratasys, USA) and Fortus 250 mc (Stratasys, USA) were used as AM machines for PC10 and ABS Plus, respectively. Regarding the process parameters of PC10 and ABS Plus, we utilized the default values provided by the manufacturer.

### 2.3. Physical Characterization

Specimens for deformation were produced in accordance with ASTM D955 using a disk-type, while specimens for surface roughness and tensile testing were prepared according to ASTM D638 Type 1 [16]. For deformation analysis, warpage was qualitatively assessed by visually inspecting the degree of lifting and distortion in the disk specimens. The R_a_ as surface roughness was measured using a stylus profilometer (SJ-410, Mitutoyo, Kawasaki-shi, Japan) over a typical evaluation length of approximately 4 mm, in accordance with ISO 4288 guidelines [17]. The measurement direction of surface roughness is perpendicular to the direction of deposition. All results of the surface roughness are displayed as the mean ± standard deviation based on three specimens.

The tensile specimens were tested at a tensile speed of 5 mm/min at room temperature using a universal testing machine (DTU-900, Daekyung Tech, Incheon Metropolitan City, Republic of Korea). The tests for surface roughness and tensile strength were performed using three samples (n = 3) for each condition. For the tensile tests, specimens were fabricated in two deposition directions, longitudinal and transverse, to compare their tensile strengths, and the strength ratio between the two directions was also evaluated. Thus, the strength ratio was defined as the tensile strength in the transverse direction divided by that in the longitudinal direction, expressed as a percentage (i.e., multiplied by 100).

## 3. Results and Discussion

### 3.1. Comparison of Shrinkage According to the Chamber Temperature

**Figure 1:**
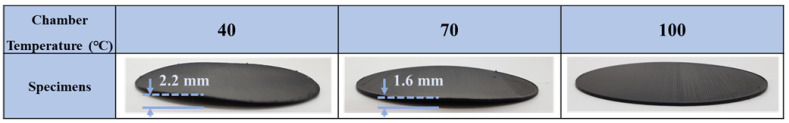
Warpage of MEXed 3DP-3A27R20 according to the chamber temperature.

First, the effect of chamber temperature on the dimensional stability of the MEXed specimens was investigated with a nozzle temperature of 240 °C, as shown in Figure 1. Specimens fabricated at chamber temperatures of 40 °C and 70 °C exhibited noticeable warping at the edges (2.2 mm and 1.6 mm at chamber temperatures of 40 °C and 70 °C, respectively). In contrast, when the chamber temperature was maintained at 100 °C, the specimens retained a stable circular shape with minimal deformation. This improvement is attributed to the reduction of thermal gradients and the mitigation of interlayer thermal contraction differences at elevated chamber temperatures, which effectively decreases residual stresses [15]. Therefore, maintaining a chamber temperature above 100 °C is essential for ensuring MEX stability.

### 3.2. Comparison of Surface Roughness According to the Nozzle Temperature

**Figure 2:**
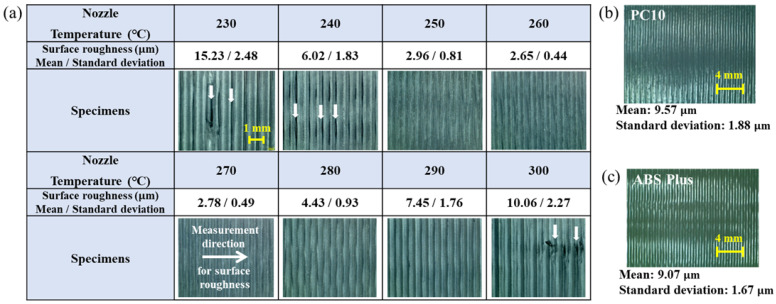
Surface roughness of MEXed (**a**) 3DP-3A27R20, (**b**) PC10, and (**c**) ABS Plus. (The arrows on the specimens with nozzle temperatures of 230 °C, 240 °C, and 300 °C indicate defects).

The effect of nozzle temperature on surface roughness was analyzed to determine the quality of interlayers at a chamber temperature of 100 °C, as shown in Figure 2a. Specimens fabricated at a nozzle temperature of 230 °C exhibited a relatively high R_a_ of 15.23 μm, whereas increasing the temperature to 250 °C significantly reduced Ra to 2.96 μm. In particular, at nozzle temperatures of 230 °C and 240 °C, the material did not melt sufficiently due to the relatively low nozzle temperature, resulting in the presence of air gaps within the specimens [18]. The lowest R_a_ of 2.65 μm was observed at 260 °C. This reduction in surface roughness represents improved adhesion of layers and fewer air gaps at the interfacial regions, which increases the effective contact area between adjacent layers [13,19]. At 270 °C, a low roughness level of R_a_ of 2.78 μm was maintained, but temperatures above 280 °C caused the extruded beads to broaden excessively due of reduced viscosity [20]. These results indicate that a nozzle temperature range of 250–270 °C is optimal for surface quality. Generally, most PCs have a nozzle temperature window at a level of 270 °C, even over 300 °C, due to their superior thermal-resistance engineering plastics [21,22]. The PC of this study was blended using bio PC, which has a relatively low nozzle temperature range from 240 °C to 270 °C [23]. Thus, this PC shows a relatively low thermal property compared to a normal PC. For a comparative assessment, we measured the surface roughness of PC10 and ABS Plus, which are widely used benchmark materials in MEX. PC10 exhibited an Ra value of 9.57 µm, while ABS Plus showed an Ra value of 9.07 µm, as shown in Figure 2b,c. Therefore, the sub-3 µm surface roughness achieved in this study demonstrates a significantly superior surface quality.

### 3.3. Comparison of Tensile Properties

**Figure 3:**
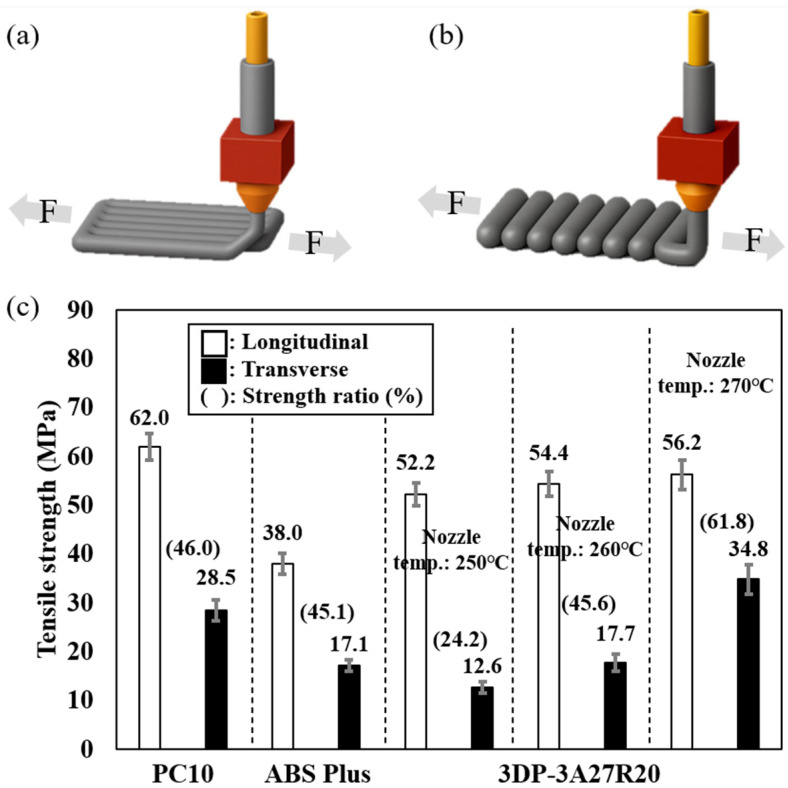
Deposition directions for (**a**) longitudinal and (**b**) transverse. (**c**) Tensile properties according to the deposition directions (Strength ratio: transverse tensile strength/longitudinal tensile strength × 100).

All specimens were fabricated with two deposition directions: longitudinal (Layers parallel to the tensile load, F.) and transverse (Layers vertical to the tensile load, F.), as shown in Figure 3a. The nozzle temperatures of 250 °C, 260 °C, and 270 °C were selected because they yielded superior surface roughness as mentioned in Section 3.2. Mechanical properties evaluation showed that, at a nozzle temperature of 270 °C, the 3DP-3A27R20 exhibited a tensile strength of 56.2 MPa in the longitudinal direction and 34.8 MPa in the transverse direction, resulting in a strength ratio of 61.8%. This indicates lower anisotropy compared to commercial PC10 and ABS Plus, which exhibit strength ratios of 46.0% and 45.1%, respectively. While the tensile strength of the longitudinal direction remained relatively consistent with increasing temperature, the tensile strength of the transverse direction increased notably with increasing nozzle temperature. Specifically, the tensile strength in the longitudinal direction measured 52.2 MPa, 54.4 MPa, and 56.2 MPa at nozzle temperatures of 250 °C, 260 °C, and 270 °C, respectively. In contrast, the tensile strength of the transverse direction improved from 12.6 MPa to 17.7 MPa and 34.8 MPa, respectively. When the nozzle temperature increased from 250 °C to 270 °C, the longitudinal tensile strength increased by approximately 0.77%, whereas the transverse tensile strength increased markedly by approximately 176%. This behavior is a commonly observed characteristic of MEXed samples [24]. Thus, 3DP-3A27R20 demonstrated excellent interlayer adhesion and reduced anisotropy in the MEX. The synergistic contribution of material design and process-parameter optimization plays a critical role in mitigating the intrinsic mechanical anisotropy associated with MEX.

## 4. Conclusions

In this study, a new PC suitable for the MEX process was adopted, and the effects of process conditions on physical properties and tensile strength anisotropy were evaluated. The results showed that maintaining a chamber temperature above 100 °C significantly reduced warping, thereby improving dimensional stability. Additionally, nozzle temperatures in the range of 250 °C to 270 °C produced excellent surface quality and enhanced interlayer bonding, resulting in high tensile strength. Notably, at 270 °C, the developed PC exhibited a strength ratio of 61.8%, which is considerably higher than those of PC10 (46.0%) and ABS Plus (45.1%), indicating significantly lower anisotropy relative to commercial materials. Overall, the developed material exhibited superior interlayer adhesion and superior physical properties relative to commercial materials, demonstrating potential to reduce the tensile strength anisotropy of MEXed components. The limitation of this paper is that the experiments were conducted using relatively simple geometries and only two deposition orientations, under a single loading condition. Future work will therefore include an expansion to various geometries, build orientations, and loading conditions to more comprehensively assess the practical reliability of MEXed components.

## Data Availability

The data presented in this study are available on request from the corresponding author. The data are not publicly available due to ongoing related research.
